# Effect of Elicitors on Morpho-Physiological Performance and Metabolites Enrichment in *Valeriana jatamansi* Cultivated Under Aeroponic Conditions

**DOI:** 10.3389/fpls.2020.01263

**Published:** 2020-09-30

**Authors:** Mahinder Partap, Pankaj Kumar, Anil Kumar, Robin Joshi, Dinesh Kumar, Ashish R. Warghat

**Affiliations:** ^1^ Cell and Tissue Engineering Laboratory, Biotechnology Division, Council of Scientific and Industrial Research (CSIR)-Institute of Himalayan Bioresource Technology, Palampur, India; ^2^ Academy of Scientific and Innovative Research, Ghaziabad, India; ^3^ Natural Product Chemistry and Process Development Division, Council of Scientific and Industrial Research (CSIR)-Institute of Himalayan Bioresource Technology, Palampur, India; ^4^ Biotechnology Division, Council of Scientific and Industrial Research (CSIR)-Institute of Himalayan Bioresource Technology, Palampur, India

**Keywords:** *Valeriana jatamansi*, aeroponic, yeast extract, methyl jasmonate, valerenic acid, ultra performance liquid chromatography, gas chromatography-mass spectrometry

## Abstract

The use of new agricultural technologies such as soilless and aeroponic cultivation systems is a valuable approach to medicinal plant production. The present study investigated the prospects of enhancing yield and secondary metabolite production in *Valeriana jatamansi* under aeroponic cultivation using elicitors, such as yeast extract and methyl jasmonate. Plants were evaluated by measuring growth parameters, photosynthetic rate, and secondary metabolites contents (on a dry weight basis). Maximum plant height (36.83 cm), leaf number (17.67), rootlet number (37.33), and rootlet length (6.90 cm) were observed at 0.5 mg/L yeast extract treatment; whereas treatment levels of 1.5 mg/L yeast extract and 150 µM methyl jasmonate resulted in maximum leaf length (6.95 cm) and leaf width (5.43 cm), respectively. Maximum photosynthetic rate (5.4053 µmol m^-2^s^-1^) and stomatal conductance (0.0656 mmol m^-2^s^-1^) were recorded at treatment levels of 0.5 mg/L and 1.5 mg/L yeast extract respectively, whereas at 150 µM methyl jasmonate treatment, transpiration rate was 0.9046 mmol m^-2^s^-1^. In aeroponic cultivation, the maximum content of valerenic acid and hydroxy valerenic acid was detected in leaf (2.47 and 8.37 mg/g) and root (1.78 and 7.89 mg/g) at treatment levels of 100 µM and 150 µM methyl jasmonate, respectively. Acetoxy valerenic acid was highest in leaf (1.02 mg/g) at 1.5 mg/L yeast extract, and in the root (2.38 mg/g) at 150 µM methyl jasmonate. Gas chromatography-mass spectrometry analysis identified twenty-eight volatile compounds in roots, of which three—isovaleric acid (6.72-50.81%), patchouli alcohol (13.48-25.31%) and baldrinal (0.74-25.26%)—were the major constituents. The results revealed that, besides roots, leaves could also be utilized as a prominent alternative source for targeted secondary metabolites. In conclusion, aeroponic cultivation offers year-round quality biomass production and ease to access subsequent roots harvest in *V. jatamansi*, to meet the demand of the pharmaceutical industries.

## Introduction

A plethora of chemically diverse natural medicinal biomolecules is derived from Himalayan herbs. *Valeriana jatamansi* Jones (Indian valerian or Tagar) is a pharmaceutically important, North-Western Himalayan medicinal and aromatic herb, found at an altitude range of 1800-3000 m above mean sea level (Mathela et al., 2005; Singh et al., 2010; Jugran et al., 2013). Valerian roots and rhizome yield 0.4-0.5% essential volatile oil, which is a valuable aromatic oil in world trade (Singh et al., 2010; Jugran et al., 2019). Due to its medicinal potential, it is commercially used in the pharma-sector for the remedy of hysteria, nervous unrest, asthma, insomnia, cholera, leprosy, and nervous disorders (Hiller and Zetler, 1996; Agnihotri et al., 2011; Bhatt et al., 2012; Joseph et al., 2016; Jugran et al., 2019). The major metabolites, such as valerenic acid (VA), acetoxy valerenic acid (AVA), and hydroxy valerenic acid (HVA) are accumulated mainly in the roots and rhizomes of the *Valeriana* species (Navarrete et al., 2006; Singh et al., 2006). Herbal companies, such as Ayumeda, Plant Therapy, Vadik Herbs, Amalth, Herbal Hills, VitaGreen, Bliss Welness, Nature’s Way, Herbal Factors, and Nature’s Health developed and marketed their herbal formulations based on high-value valerenic acid content (0.8%). A minimum content of 3.0 mg/g valerenic acid has been recommended for high-quality valerian for herbal formulations on a commercial scale (Bos et al., 1998). In India, the market price of dried rhizome and roots of *V. jatamansi* valued at Rs. 440 per kg, and the estimated annual trade is 1000-2000 metric tons (Goraya and Ved, 2017).

The industrial market demand for *Valeriana* phytochemicals is relying entirely on its wild habitat and traditional agro-practices (Fonseca et al., 2006; Hayden, 2006). Due to the huge demand-supply gap, the use of Hi-Tech/modern agro-practices, such as hydro-aeroponic offers an alternative solution for quality biomass production, as per the industrial standards (Dorais et al., 2001; Hayden, 2006; Tabatabaei, 2008; Thakur et al., 2019). According to global market size, aeroponic farming was valued at USD 126.2 million in 2017, and expected to grow to USD 759.4 million by 2025, with a compound annual growth rate (CAGR) of 25.5% (Anonymous, 2019). Aeroponic cultivation not only reduces the crop duration, but also can enhance the metabolites content through elicitation strategies (Huttner and Bar-Zvi, 2003; Prasad et al., 2012).

Elicitors, such as methyl jasmonate and yeast extract, act as signaling molecules, recognized by elicitor‐specific receptors on the plant cell membrane, and induce transcriptional activation of genes involved in the biosynthesis of secondary metabolites (Cui et al., 2012; Torkamani et al., 2014; Halder et al., 2019). However, for efficient and effective elicitation processes, optimization of different parameters, such as type, concentration, exposure duration, and treatment schedule of elicitors are the foremost requirements (Halder et al., 2019), but to date no report is published on the elicitors (such as methyl jasmonate and yeast extract) mediated enhancement of plant growth and secondary metabolites content in aeroponically-cultivated *V. jatamansi*. Hence, we conceived the present study to develop a simple yet feasible and robust protocol for enhanced production of plant growth and secondary metabolites.

## Materials and Methods

### Plant Material

Nursery grown plants of *V. jatamansi* (sizes 18 to 20 cm height) were used as experimental material, and procured from the Agrotechnology Division, CSIR-Institute of Himalayan Bioresource Technology, Palampur, Himachal Pradesh, India. All the experiments were conducted from September 2018 to December 2018 under an aeroponic facility of CSIR-IHBT, Palampur.

### Aeroponic Cultivation System

The aeroponic facility consisted of a fully automatic control unit for fertigation, nutrigation, and other parameters, such as photoperiod, temperature regulation, and relative humidity. The system contained reservoir tanks of 1000 L capacity for the nutrient solution, which were automatically refilled with reverse osmosis water. UV lamps (Ecostream UV, Ace Hygiene Products Private Limited, Mumbai, India) were installed in the system for the sterilization of nutrient solution. Aeroponic cultivation chambers had dimensions 1.5 m long x 0.5 m wide x 0.4 m depth with the provision for complete darkness inside to protect the root zone from light. The chambers consisted of a horizontal platform with holes (75 mm diameter) for plant cultivation at a spacing range of 15 x 25 cm. Corrosion resistance spray jets (4LPH-Netafilm) with a fine orifice (0.025”) were used for intermittently spraying nutrient solution mist (droplet size 50 micron) through a high-pressure accumulator pump (1.0 HP, Pedrollo, Italy). Air bubbler units (Crompton, Mumbai, India) were used to provide oxygenated nutrients solution to roots. Elicitors treatment was provided by spray jet *via* expansion ports joined to cultivation chambers for elicitation experimentation. Electrical conductivity (EC) and pH of nutrient solution were monitored using EC and pH sensors (Hanna Instruments, Padova, Italy).

### Nutrient Medium and Sterilization

**Figure 1:**
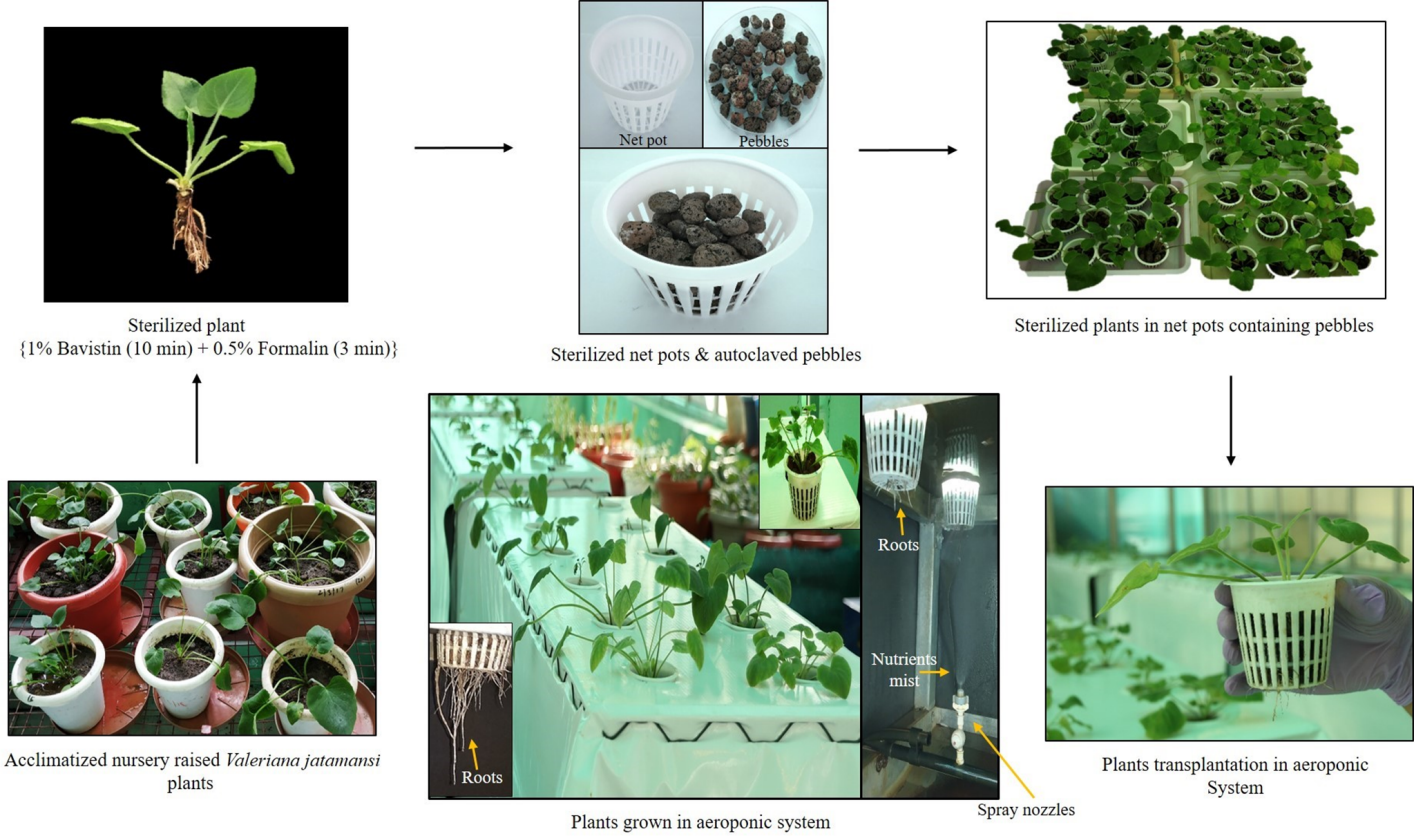
Aeroponic cultivation process and protocol for *V. jatamansi*.

Nutrient medium (Hoagland No. 2 Basal Salt Mixture, TS1094, Hi-Media, Mumbai, India) was used for the aeroponic cultivation of *V. jatamansi.* Basal salt mixture (0.96 g/L) was dissolved in the reverse osmosis water, properly homogenized, and pH was adjusted to 6.8-7.0 using 1.0 N NaOH (sodium hydroxide) and 1.0 N HCl (hydrochloric acid). Plants and net pots were sterilized using 1.0% bavistin (Hi-Media, Mumbai, India) and 0.5% formalin solution (Hi-Media, Mumbai, India) for 10 min and 3 min, respectively. Pebbles and soil were sterilized by autoclave at 121°C, 103 KPa pressure for 30 min. After sterilization, plants were anchored in the plastic net pots containing pebbles and transplanted to aeroponic chambers (**Figure 1**). For soil pot cultivation, the physicochemical properties of the soil, in which *V. jatamansi* were grown have been already reported by Singh et al., 2010; Kaundal et al., 2018; Dhiman et al., 2020. The soil was silty clay loam in texture, slightly acidic (pH - 6.3), organic carbon (2.3%), available nitrogen (198 kg ha^−1^), available phosphorous (23 kg ha^−1^) and available potassium (538 kg ha^−1^) content (Singh et al., 2010).

### Elicitors Selection, Preparation, and Screening

In the present investigation, methyl jasmonate and yeast extract were selected for the elicitation studies. Methyl jasmonate (MJ) (Sigma Chemical Co., St. Louis, USA) was dissolved in 96% (v/v) ethanol (Hi-Media, Mumbai, India), and prepared as a stock (1.0 M) solution. The solution was filtered through 0.22 µm filter (Sartorius, Germany). A 40x stock solution of yeast extract (YE) (Hi-Media, Mumbai, India) was prepared in distilled water, and then filter-sterilized. Preliminary screening studies were carried out using different concentrations of YE (0.25, 0.5, 0.75, 1.0, 1.25, 1.50, 1.75 and 2.0 mg/L), and MJ (25, 50, 75, 100, 125, 150, 175 and 200 µM) in basal salt nutrient medium for 10 days. Based on morphological observations, such as plant height, leaf number, leaf length, leaf width, rootlet length, and rootlet number; best elicitor concentrations of YE (0.5, 1.0 and 1.5 mg/L) and MJ (50, 100 and 150 µM) were finally selected, for further experimentation.

### Experimental Set-Up

**Figure 2:**
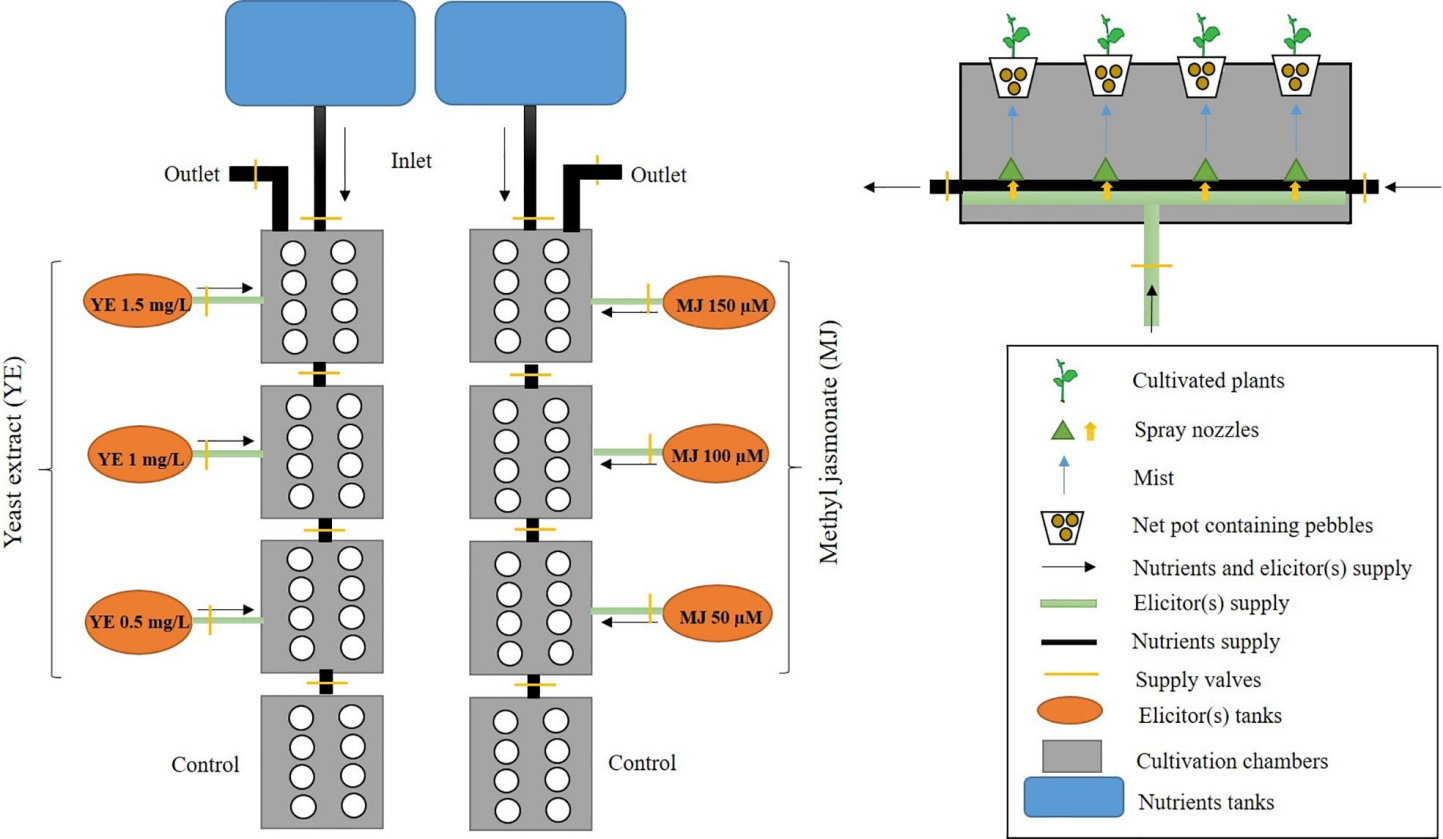
Experimental design of aeroponic cultivation system for *V. jatamansi*.

Two cultivation systems (soil-based pot and aeroponic) with one hundred and ninety-two plants (ninety-six plants per cultivation) were used in the present investigation, to study the effect of YE and MJ on morpho-physiological performance, and secondary metabolites augmentation in *V. jatamansi*. Ninety-six plants were planted in eight growth boxes having twelve-plant cultivation capacity per box in the aeroponic system. Out of 96 cultivated plants, 72 plants (12 per each treatment) were treated with YE (0.5, 1.0 and 1.5 mg/L) and MJ (50, 100, and 150 µM), respectively, while 24 plants were cultivated without elicitor treatments, considered as control. Roots were irrigated using the nutrient solution with 10 minutes intermittent spray duration. Six elicitor concentrations (three of each) of YE (0.5, 1.0 and 1.5 mg/L), and MJ (50, 100, and 150 µM) irrigated the root zone with 30 minutes spray duration, three times in a day (**Figure 2**). To maintain the proper plant growth and development, the EC range was standardized and maintained, i.e. 2.0-2.8 mS cm^-1^. As mentioned in the supplementary file (**Figure S1**), for the initial growth phase, EC was maintained at 2.0 (days 1-35) and 2.5 (days 36-63); at harvest stage, EC was 2.8 (days 64-98). The aeroponic system temperature (25 ± 2°C), relative humidity (65-70%), photoperiod (16h light/8h dark), pH (6.8-7.0), nutrient solution temperature (10-11°C) using chiller (Voltas Limited, Mumbai, India), and photosynthetic photon flux density (150 µmol m^-2^ s^-1^) using PAR-lamp (Bajaj Electricals, Mumbai, India) were maintained throughout the experiment (supplementary data file; **Figure S1**). Water, nutrients, and elicitors solution loss were monitored daily and the tank was filled at weekly intervals. A similar experimental set up was also followed for the soil-based pot cultivation system.

### Morpho-Physiological Assessment

Morphological characters, such as plant height (cm), leaf number, leaf length (cm), leaf width (cm), rootlet number, and rootlet length (cm) were recorded at weekly intervals. Morphological traits were represented in a box plot, and coefficient of variation (CV) was used to interpret variation within or among studied traits. Physiological performance parameters, such as photosynthetic rate, stomatal conductance, and transpiration rate of the mid-lamina portion of the fully expanded young leaves were measured in treated and control plants, using a portable infra-red gas analyzer (IRGA; model Li-6400; Li-Cor, Lincoln, USA). The following conditions were maintained: the flow rate 500 µmol s^-1^; leaf area 6.0 cm^2^; cabinet air temperature 20°C and relative humidity 67-68%. The reference CO_2_ concentration was set according to the internal CO_2_ concentration of the system.

### Sample Preparation

Dried tissue samples of *V. jatamansi* (leaf and root) were pulverized into a fine powder using mortar and pestle, separately. 500 mg of dried powder of each sample was macerated in 5.0 mL of HPLC grade absolute methanol (Merck Life Science Private Limited, Mumbai, India) for ultra-performance liquid chromatography (UPLC) quantification, and dried root samples (500 mg) macerated in HPLC grade dichloromethane (5.0 mL) for gas chromatography-mass spectrometry (GC-MS) analysis. All the samples were sonicated for 30 min at 40°C, and centrifuged at 2000 rpm for 2 min. The collected supernatant was filtered through a 0.22 μm pore size NEXFLO polyvinylidene syringe filters (Moxcare Products Incorporation, Haryana, India), for the quantification of valerenic acid and its derivatives (AVA and HVA) (Merck Life Science Private Limited, Mumbai, India) using the UPLC technique and identification of volatile compounds using GC-MS analysis. Concentration (1.0 mg/mL) of reference standards (AVA, HVA, and VA) were prepared with HPLC grade methanol for UPLC analysis. 500 µg/mL concentration of the standard mixture (equal volume of each standard) was made, and serial dilution was prepared as 50, 100, 200, and 400 µg/mL, respectively. The concentration of test samples (100 mg/mL) was prepared for phytochemical analysis.

### Ultra Performance Liquid Chromatography (UPLC) Quantification

Valerenic acid and its derivatives (HVA and AVA) analysis were performed on Waters Acquity UPLC-H class system (Waters Corporation,** **Milford, Massachusetts, United States) equipped with eλ photodiode array detector (PDA), an autosampler, 600 controller™ pump with online degasser, column heater, and a binary solvent manager. Water BEH C18 column (2.1 x 100 mm, 1.7 µm particle size) was used, fitted with a suitable guard column. Mobile phase A was 0.1% phosphoric acid in water and B in acetonitrile (Merck Life Science Private Limited, Mumbai, India). The gradient program was 0 min A 40%, 6.0 min A 5.0%, 8.5 min A 5.0% and 12 min A 40% at a flow rate of 0.25 mL/min. The injection volume of 2.0 µL was used for simultaneous identification and quantification analysis. The calibration curves for AVA, HVA, and VA were generated using peak area and concentration.

### Gas Chromatography-Mass Spectrometry (GC-MS) Analysis for the Identification of Volatile Compounds

Volatile constituents were analyzed by Shimadzu GC-MS QP 2010 (Shimadzu Corporation, Kyoto, Japan) fitted with AOC 5000 autoinjector system, and operated in the EI mode at 70 eV, equipped with ZB-5MS column, (Phenomenex, USA). The following conditions were maintained: the initial temperature at 70°C for 3.0 min, ramped at 4.0°C/min to 220°C, and held for 5.0 min. Helium gas was used as a carrier at a flow rate of 1.05 mL/min. The sample volume 1.0 μL was injected in split mode ratio (1:10). The injector and detector temperature were set at 240°C and 250°C, respectively. The identity of each compound was assigned by comparing their relative retention index (RRI) relative to the n-alkane mixture (C_9_-C_24_). Compounds were also characterized by a comparison of their spectra with those available from MS libraries such as National Institute of Standards and Technology (NIST) database (McLafferty, 2000).

### Statistical Analysis

All the experimental results were expressed as mean ± standard error using statistical software, SPSS 25.0.0 (Statistical Program for Social Sciences, SPSS Corporation, Chicago, USA). One-way analysis of variance (ANOVA) with Duncan and Dunnett’s Post Hoc multiple comparison tests (*p* ≤ 0.05) was performed. Morphological traits were assessed in a box plot analysis using PAleontological STatistics (PAST) Version 3.25 (means and 0.95 confidence intervals). The box plot showed 25^th^ - 75^th^ percentile; center line, median; whiskers, full data range, i.e., minimum value, lower quartile (Q1), median, upper quartile (Q3), maximum value and interquartile range (IQR = Q3 - Q1), which covers the central 50% of the data. The coefficient of variation (CV) was calculated using the formula [CV% = (standard deviation/mean) × 100] to interpret variation within or among morphological traits.

## Results and Discussion

### Morphological Traits Analysis

**Figure 3:**
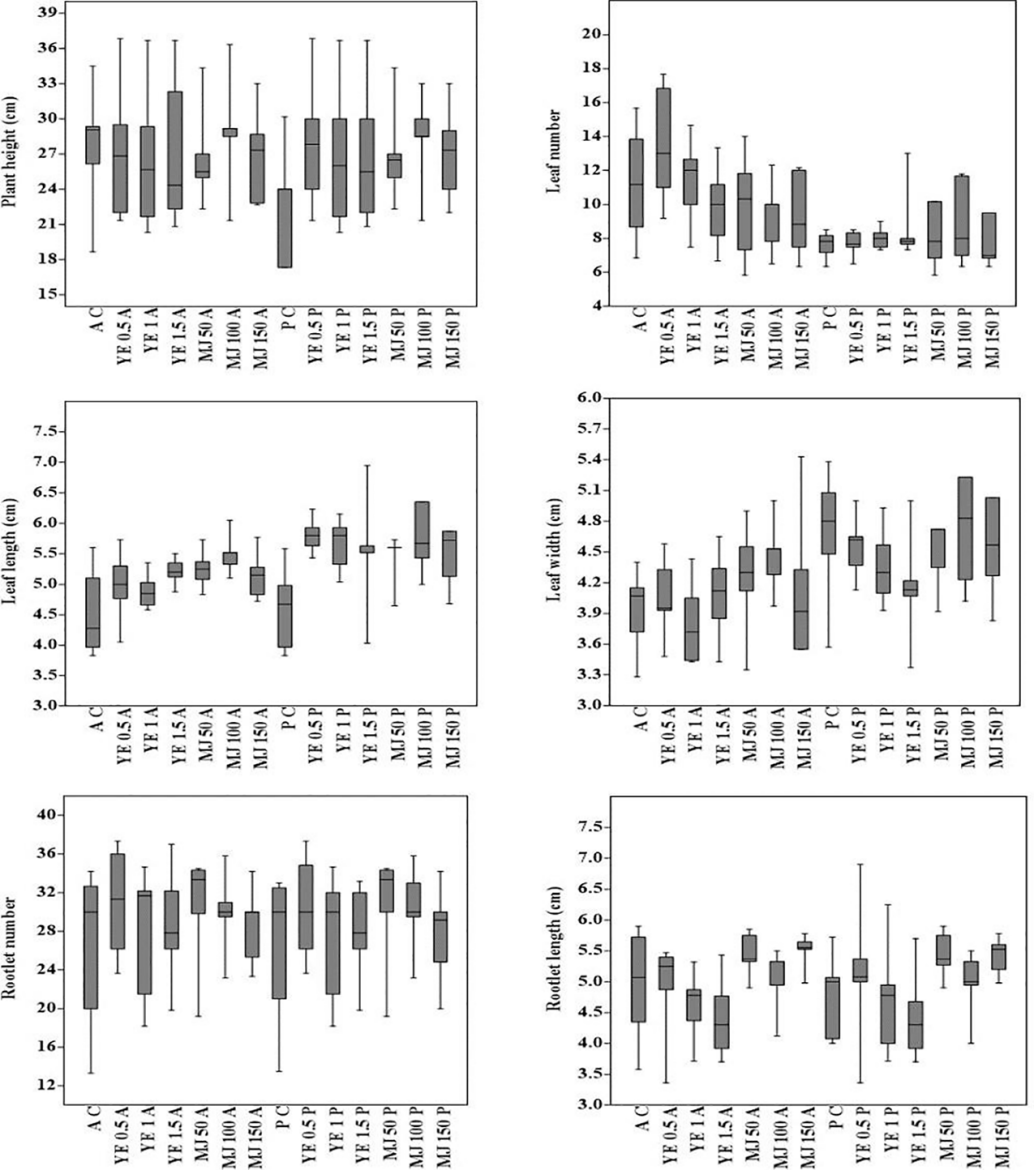
Box plot analysis (means and 0.95 confidence intervals), showing the minimum, first quartile, median, third quartile and maximum values for comparison of morphological characters of plants cultivated under different systems [AC, aeroponic control; A, aeroponic; YE, yeast extract (0.5, 1 and 1.5 mg/L); MJ, methyl jasmonate (50, 100, and 150µM); PC, pot control; P, pot]. To interpret variation coefficient of variation (CV) was calculated using formula [CV % = (standard deviation/mean) × 100].

**Figure 4:**
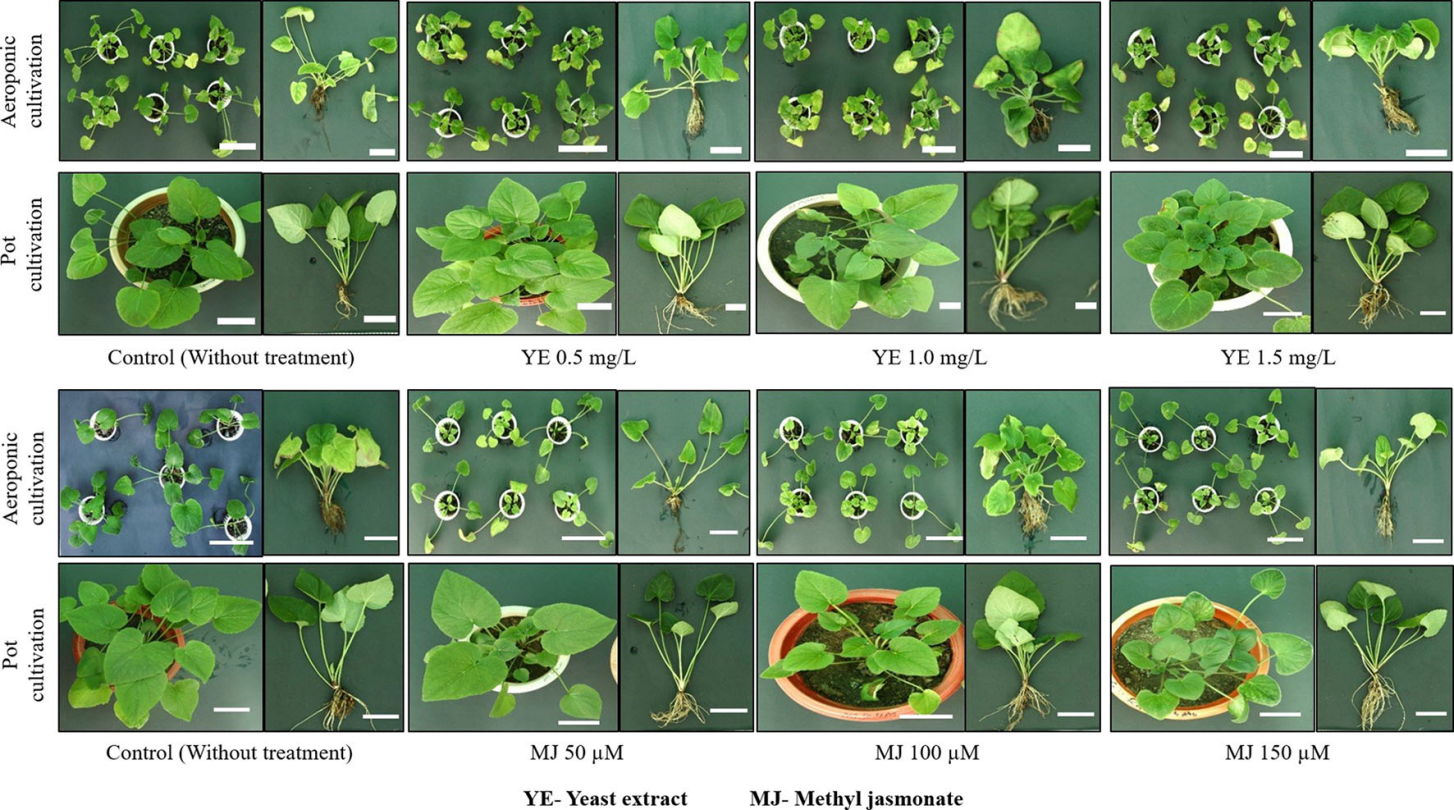
Comparative morphological assessment of aeroponic and pot cultivated *V. jatamansi*. Scale bar = 10 cm.

Morphological traits varied in terms of plant growth parameters, i.e. the coefficient of variation in aeroponic and pot cultivations ranged from 1.84% (leaf length) to 17.27% (leaf number). In both cultivation systems, the coefficients of variation for the studied traits were: plant height 12.12%, leaf width 2.94%, rootlet number 15.14% and rootlet length 7.47%. In aeroponic and pot cultivations, CV values interpreted in percentage (%) were, respectively: plant height 12.62, 11.99; leaf number 22.55, 11.89; leaf length 4.05, 2.42; leaf width 4.48, 5.93; rootlet number 15.23, 15.11 and rootlet length 7.98, 7.28. For each morphological character, 25^th^-75^th^ percentile of the quartiles were drawn using the box. The median has been shown with a black horizontal line and minimal and maximal values were represented as short horizontal lines i.e. whiskers. The whisker interval was the 95 percent confidence interval for the estimate of the mean, based on the standard error. Plant growth parameters showed a different reaction to YE and MJ elicitors treatment. In aeroponic cultivation, maximum plant height, leaf number, leaf length, rootlet number, and rootlet length were observed in 0.5 mg/L YE, while maximum leaf width was observed in 1.5 mg/L YE treatment. Whereas, in methyl jasmonate treatment, maximum plant height, leaf length, and rootlet number were observed in 100 µM, while maximum leaf number, leaf width, and rootlet length were observed in 50 µM, 150 µM and 50 µM concentration, respectively **(** **Figure 3** **)**. The results of the study concluded that, 0.5 mg/L YE treatment is the most suitable elicitor concentration for *V. jatamansi* plants cultivated under aeroponic system. Overall, in the present study, no morphological aberrations were detected when plants were treated with different concentrations of YE and MJ, compared to control **(** **Figure 4** **)**. Mu et al. (2009) also reported that appropriate doses of biotic and abiotic elicitor resulted in a positive effect on the plant growth and development in *Lycoris chinensis*. The present study also highlighted that an increased trend of plant growth was observed with the increased EC of the nutrient solution (i.e. 2.0-2.8 mS cm^-1^). As per available literature, standardization of EC of the nutrient solution is necessary for optimal plant growth and development stages in hydro-aeroponic cultivation (Tabatabaei, 2008; Ding et al., 2018; Thakur et al., 2019). *V. jatamansi* is a temperate plant and prefers cold winters and mild summers for its optimum growth with temperatures of 15-25°C (Dhiman et al., 2020). The optimal root growth of *V. jatamansi* has been found to occur at a soil temperature of 8-12°C. Therefore, in the present study, the nutrient solution temperature was standardized at 10-11°C using a chiller for optimal root growth. Though, as per the available literature; root zone temperature is the important factor required for the improvement of root growth rate (Solfjeld and Johnsen, 2006; Sakamoto and Suzuki, 2015; Thakur et al., 2019). Sadeghi et al. (2014) reported aeroponic cultivation facilitates, improved roots growth by preventing peripheral friction, and decreased the energy consumption down to 70% as compared to soil cultivation. Nutrient recipe, nutrient management strategy, elicitors treatment (YE and MJ), reduced roots peripheral friction, and controlled environmental conditions, resulted in improved plant growth and development with no morphological abnormalities. In the present finding, subsequent harvesting of metabolite enriched roots biomass without sacrificing *V. jatamansi* plants was also possible.

### Photosynthetic Performance

An infra-red gas analyzer was used to measure photosynthetic rate, stomatal conductance, and transpiration rate in treated versus control plants. The maximum photosynthetic rate (5.4053 and 4.7703 µmol m^-2^s^-1^) was observed in elicitor treated plants (0.5 mg/L YE and 50 µM MJ), cultivated under aeroponic as compared to control. However, pot treated plants showed maximum photosynthetic rate (4.6043 and 2.9125 µmol m^-2^s^-1^) in 0.5 mg/L YE and 150 µM MJ, as compared to control, respectively. Elicitor treatment of YE (1.5 mg/L) resulted in higher values of stomatal conductance (0.0415 mmol m^-2^s^-1^) and transpiration rate (0.4759 mmol m^-2^s^-1^), whereas in MJ (150 and 50 µM) treatment, maximum stomatal conductance (0.0435 mmol m^-2^s^-1^) and transpiration rate (0.3922 mmol m^-2^s^-1^) were observed in aeroponic cultivation system, respectively. In pot cultivation, YE and MJ (1.5 mg/L and 150 µM) treatment showed maximum stomatal conductance (0.0656 and 0.0309 mmol m^-2^s^-1^) and transpiration rate (0.8503 and 0.9046 mmol m^-2^s^-1^) as compared with the control condition, respectively (**Table 1**).

**Table 1:** Effect of cultivation systems and elicitors treatment on the physiological performance of *V. jatamansi*.

Physiological parameters	Aeroponic cultivation							Pot cultivation						
	Control	Yeast extract (mg/L)			Methyl jasmonate (µM)			Control	Yeast extract (mg/L)			Methyl jasmonate (µM)		
		0.5	1.0	1.5	50	100	150		0.5	1.0	1.5	50	100	150
Photosynthetic rate (µmol m^−2^s^−1^)	2.6742 ± 0.0443^cd^	5.4053 ± 0.0430^i^	2.6492 ± 0.0626^c^	3.9057 ± 0.0438^f^	4.7703 ± 0.0359^h^	3.7313 ± 0.0183^ef^	3.6306 ± 0.0864^e^	2.2829 ± 0.085^b^	4.604 ± 0.0527^gh^	1.42108 ± 0.0185^a^	4.4871 ± 0.1099^g^	2.4546 ± 0.0772^bc^	2.4901 ± 0.0996^bc^	2.9125 ± 0.0714^d^
Stomatal conductance (mmol m^−2^s^−1^)	0.0197 ± 0.0006^bc^	0.0339 ± 0.0003^g^	0.0268 ± 0.0005^def^	0.0415 ± 0.0004^h^	0.0283 ± 0.0002^defg^	0.0220 ± 0.0002^bcd^	0.0435 ± 0.0042^h^	0.0255 ± 0.0013^cdef^	0.0299 ± 0.0003^efg^	0.0189 ± 0.0001^b^	0.0656 ± 0.0015^i^	0.0107 ± 0.0003^a^	0.0239 ± 0.0012^bcde^	0.0309 ± 0.0007^fg^
Transpiration rate (mmol m^−2^s^−1^)	0.2939 ± 0.0094^bc^	0.4656 ± 0.0034^f^	0.3793 ± 0.0074^de^	0.4759 ± 0.0043^f^	0.3922 ± 0.0032^de^	0.3041 ± 0.0024^c^	0.1570 ± 0.0016^a^	0.3608 ± 0.0158^d^	0.4164 ± 0.0033e	0.2626 ± 0.0015^b^	0.8503 ± 0.0195^g^	0.1812 ± 0.0045^a^	0.2923 ± 0.0011^bc^	0.9046 ± 0.0100^h^

Data represents mean ± SE (n = 3). Different superscripts letters in a row specify statistically significant difference between the means (p ≤ 0.05, Duncan Multiple Range Test).

In the present investigation, the elicitors treatment resulted in the improvement of photosynthetic rate in aeroponic cultivated *V. jatamansi* plants. White et al. (2016) reported, that photosynthesis rate elevated with CO_2_ concentration, typically limits the growth under ambient CO_2_ level. However, the increased photosynthetic rate and yield were reported, when the plants were grown under an aeroponic polyhouse, where CO_2_ concentration was higher than the ambient condition. Hasanah and Sembiring (2018) concluded that, elicitors application in soybean cultivars resulted in the improvement of photosynthetic performance, i.e. high chlorophyll content and stomatal density. Recently, Hassini et al. (2019) also observed the positive response of elicitors on plant physiology concerning water relations and mineral nutrition in broccoli. From the present findings, application of methyl jasmonate and yeast extract treatments, resulted in increased photosynthetic rate that was possibly due to higher chlorophyll content, nutrient availability, elevated CO_2_, proper aeration to root zone, and increased leaf surface area (as leaves are the primary source for photo-assimilation). Furthermore, no deficiency and aberrations symptoms were observed in terms of physiological performance of *Valeriana* plants, in elicitors mediated aeroponic cultivation.

### Quantification of Valerenic Acid and Its Derivatives

**Figure 5:**
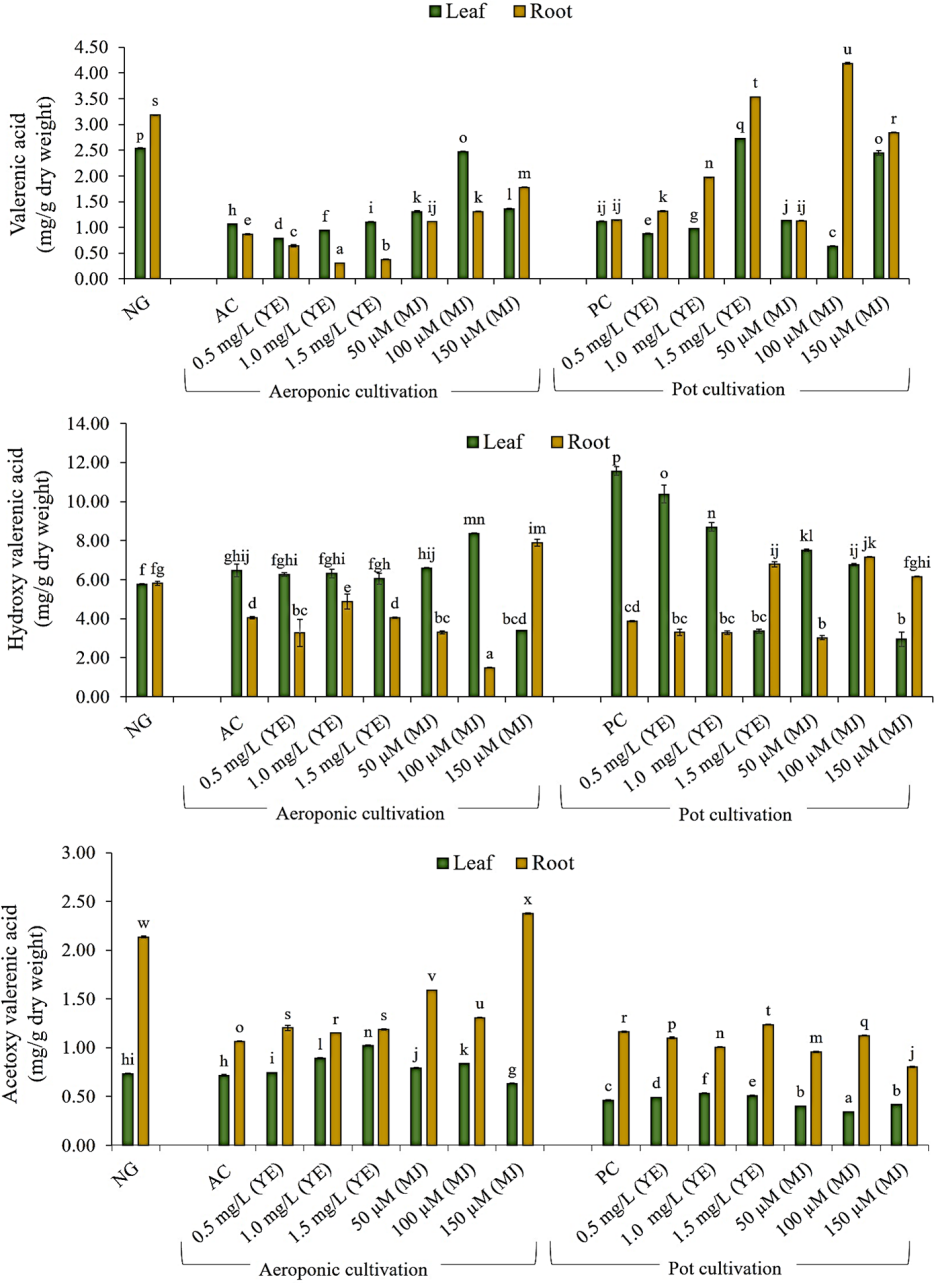
Quantification of valerenic acid and its derivatives in leaf and root of *V. jatamansi* cultivated in aeroponic and pot systems (NG, nursery grown; AC, aeroponic control; PC, pot control; YE, yeast extract; MJ, methyl jasmonate). Data bars represent mean ± SE (*n* = 3). Different superscript letters on bars represent statistically significant difference between the mean values (*p* ≤ 0.05, Duncan Multiple Range Test).

**Figure 6:**
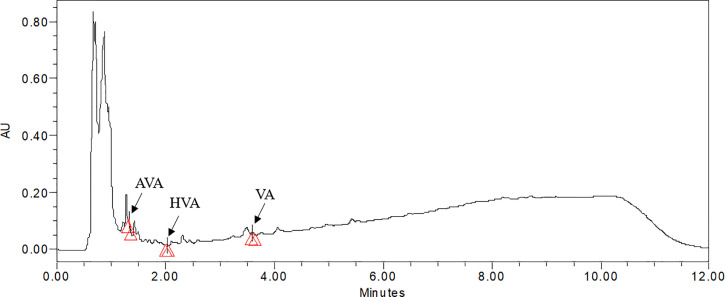
UPLC chromatogram of 100 µM methyl jasmonate treated sample of *V. jatamansi* cultivated in aeroponic system (AVA, acetoxy valerenic acid; HVA, hydroxy valerenic acid; VA, valerenic acid).

In the present investigation, major sesquiterpenoids (AVA, HVA, and VA) of *V. jatamansi* were analyzed using the UPLC technique. The calibration curve, linearity range, regression equation, correlation coefficient, and chromatogram of standard marker compounds (AVA, HVA, and VA) were given in supplementary **Table S1**. The UPLC chromatograms of samples (leaf and root) were represented in supplementary data files (**Figures S2–S31**). The maximum content of VA and HVA were observed in leaf (2.47 and 8.37 mg/g DW), and root (1.78 and 7.89 mg/g DW) of aeroponically cultivated plants treated with MJ (100 µM and 150 µM), respectively (**Figures 5**, **6** **)**. The maximum content of AVA (2.38 mg/g DW) was detected in root treated with 150 µM of MJ, while in 1.5 mg/L YE treatment, a higher content of AVA (1.02 mg/g DW) was detected in leaf, as compared to controls. However, treatment of YE (1.5 mg/L) and MJ (100 µM) resulted in higher VA content accumulation in leaf (2.72 mg/g DW) and root (4.19 mg/g DW) under the pot cultivation system, respectively. Maximum HVA content (7.16 mg/g DW) was detected in root treated with MJ (100 µM) concentration, while the leaf of control condition accumulates overall maximum content of HVA (11.56 mg/g DW). AVA content was found highest in the root (1.24 mg/g DW) and leaf (0.53 mg/g DW) of YE treatment (1.5 and 1.0 mg/L), respectively. Whereas, a significant amount of VA, HVA, and AVA content was also detected in nursery-grown leaf (2.54, 5.75 and 0.73 mg/g DW) and root (3.18. 5.81 and 2.14 mg/g DW). Aeroponic and soil-based pot cultivations showed comparable metabolites content to nursery-grown plants when treated with elicitors (**Figure 5**). Effective use of yeast extract (0.5, 1.0, and 1.5 mg/L), and methyl jasmonate (50, 100, and 150 µM) in the aeroponic system increased the number of metabolites at a faster rate.

Cui et al. (2012) observed a threefold increase in valtrate content using 100 mg/L MJ treatment, in the adventitious root culture of *V. amurensis.* Similarly, Torkamani et al. (2014) reported increased valerenic acid production (two times higher) in *V. officinalis* hairy root culture with MJ (100 μM) treatment. In the present investigation, elicitation resulted in a significant increase of VA, HVA, and AVA content, not only in the root (1.78, 7.89, and 2.38 mg/g DW) but most prominently in the leaf (2.47, 8.37 and 1.02 mg/g DW), for the first time. In aeroponic cultivation, optimized temperature, humidity, chilling effect, aeration to root zone and root zone temperature (10°C) conditions resulted in augmentation of metabolite contents (Stoner, 1983; Soffer and Burger, 1988; Pagliarulo and Hayden, 2000; Sakamoto and Suzuki, 2015; Thakur et al., 2019). The present results highlight that elicitors treated plants produced higher amounts of key secondary metabolites, i.e. VA, HVA, and AVA in *V. jatamansi* plants cultivated under aeroponic conditions.

### Volatile Compounds Estimation

In the present study, GC-MS analysis identified twenty-eight volatile compounds in roots of elicitors treated and control plants. The total identified percentage of all volatile compounds ranged from 48.07 to 92.86% in both cultivations system (**Table 2**). The major compounds identified ranged from 6.72 to 50.81% (isovaleric acid), 13.48 to 25.31% (patchouli alcohol), 0.74 to 25.26% (baldrinal), 2.67 to 18.26% (4-aceto-oxycinnamic acid), 0.25 to 8.33% (β- gurjunene), 0.38 to 6.67% (caryophyllene), 1.02 to 6.44% (juniper camphor), 1.67 to 5.08% (allyl isovalerate), 0.93 to 4.95% (α- guaiene), 0.27 to 4.46% (octadecanoic acid), 0.71 to 4.31% (γ- patchoulene), 0.61 to 3.89% (diacetone alcohol), 1.03 to 3.84% (α- santalene), and 0.66 to 3.13% (viridiflorol).

**Table 2:** GC-MS analysis for the identification and quantification of volatile compounds in *V. jatamansi* roots.

Compounds	RRI (Relative retention indices)*	Nursery grown	Aeroponic cultivation							Pot cultivation							Range (%)
			Control	Yeast extract (mg/L)			Methyl jasmonate (µM)			Control	Yeast extract (mg/L)			Methyl jasmonate (µM)			
				0.5	1.0	1.5	50	100	150		0.5	1.0	1.5	50	100	150	
Isovaleric acid	839**	–	22.76	–	–	38.53	40.46	6.72	–	49.77	43.31	37.19	50.81	9.30	–	–	6.72-50.81
3-Methyl valeric acid	946**	0.36	0.93	0.66	–	1.43	2.40	3.14	–	–	–	0.58	1.04	–	–	1.82	0.36-2.40
Diacetone alcohol	1138	0.61	1.12	–	–	1.47	3.89	1.79	–	1.77	1.12	1.68	2.06	1.37	–	–	0.61-3.89
α-Santalen	1316	–	–	1.31	3.84	1.03	–	–	–	–	–	–	–	–	1.48	–	1.03-3.84
α-Guaiene	1326	–	1.10	–	4.95	1.61	0.93	–	–	–	–	–	–	1.29	3.44	–	0.93-4.95
β-Gurjunene	1329	–	–	4.52	8.33	–	–	0.25	–	–	–	–	–	–	–	–	0.25-8.33
Azulene	1332	–	–	1.06	–	–	–	–	1.59	–	–	–	–	0.99	1.71	–	0.99-1.71
Seychellene	1339	0.78	–	–	–	2.37	2.44	1.38	1.74	–	–	–	–	1.54	2.99	1.18	0.78-2.99
Caryophyllene	1343	–	–	2.00	6.67	1.93	–	–	–	–	–	0.38	–	0.70	2.89	–	0.38-6.67
α-Patchoulene	1361	0.45	2.95	1.91	2.84	2.19	0.57	0.75	1.31	–	–	–	–	1.15	2.18	0.68	0.45-2.95
Allyl isovalerate	1394	–	–	–	–	1.83	2.39	–	–	2.66	4.96	5.08	1.67	–	–	–	1.67-5.08
γ-Patchoulene	1407	–	1.89	2.49	4.31	–	–	–	–	0.86	0.71	2.22	1.05	–	–	–	0.71-4.31
Selinene	1418	0.23	–	1.03	–	–	–		0.48	–	–	0.51	–	0.46	–	–	0.23-1.03
Baldrinal	1430	18.01	8.09	25.26	11.05	–	–	16.38	8.32	–	–	1.66	0.74	23.44	6.35	11.87	0.74-25.26
α-Humulene	1460	–	–	–	1.58	0.69	–	–	–	–	–	–	–	–	0.72		0.69-1.58
Juniper camphor	1469	–	1.65	5.48	8.65	–	2.10	1.75	1.02	–	–		–	–	6.44	3.21	1.02-6.44
Eudesmol	1494	–	–	–	–	–	0.61	–	–	0.39	0.34		0.42		–	–	0.34-0.61
Panasinsene	1495	–	0.89	–	–	1.17	0.84	–	–	–	–	–	–	0.73	–	–	0.73-1.17
Globulol	1581	0.80		0.65	–	0.68	0.88	–	–	0.97	0.52		0.52		–	1.02	0.52-1.02
Viridiflorol	1598	–	1.61		1.86	3.13	2.26	–	1.93	1.77	1.46	1.59	1.28	1.17	–	0.66	0.66-3.13
Thujopsan-2-alpha-ol	1611	0.37	–	–	–	–	–	0.44	–	0.38	–	0.47	–	–	–	0.34	0.34-0.47
Thujopsanone	1667	0.16	–	0.61	–	0.54	–	0.22	–	–	–	0.41	–		–	–	0.16-0.61
Longipinanol	1684	0.19	0.75	–	–	1.03	0.96	0.19	–	0.59	0.52		0.65	0.32	–	–	0.19-1.03
Patchouli alcohol	1719	14.92	18.49	20.70	25.31	23.42	21.41	17.97	18.33	15.68	13.48	20.34	15.65	20.37	23.03	24.44	13.48-25.31
Hexadecanoic acid	1913	–	1.08	–	–	2.79	1.36	–	–	1.81	–	2.03	1.53	–	–	–	1.08-2.79
Isopropyl valerate	2015	–	–	–	–	0.43	0.69	–	–	0.80	–	0.49	0.72	0.43	–	–	0.43-0.80
Octadecanoic acid	2122	–	4.46	–	–	1.38		0.55	–	–	1.28	1.38	1.05	0.27			0.27-4.46
4-Acetooxycinnamic acid	2134	18.26	2.67	13.35	13.47	2.81	–	16.34	13.35	3.67	–	5.14	4.06	15.56	8.18	15.77	2.67-18.26
Total identified (%)		55.14	70.44	81.03	92.86	90.46	84.19	67.87	48.07	81.12	67.7	81.15	83.25	79.09	59.41	60.99	48.07-92.86

*Relative retention indices relative to C_9_-C_24_ n-alkane mixture.

**Relative retention indices values as per literature cited by Javidnia et al., 2006; (-) Not detected.

However, no prior information was available on elicitors effect for the identification of volatile compounds in *V. jatamansi* under aeroponic cultivation. Bhatt et al. (2012) reported twenty volatile compounds using GC, and GC-MS analysis in *V. jatamansi* extract, and the major compounds were patchouli alcohol, seychellene, α‐guaiene, and α‐humulene, etc. Liu et al. (2013) identified twenty-seven volatile compounds with the total identified percentage of 94.8% in the essential oil extract of *V. jatamansi*, with the maximum percentage of patchoulol (24.3%), isovaleric acid (12.9%), α-guaiene (8.7%), and 3-methylvaleric acid (8.4%). However, for the first time, the present investigation reported a higher percentage of isovaleric acid (50.81%), patchouli alcohol (25.31%), baldrinal (25.26%), 4-aceto-oxycinnamic acid (18.26%) and β- gurjunene (8.33%). In both cultivation systems, the maximum total volatile identified compounds (92.86%) was reported in 1.0 mg/L YE, while in the case of methyl jasmonate treatment 84.19% was found in 50 µM MJ. Overall, nutrient media, elicitors treatment, elicitors concentrations, and system environmental conditions could be responsible factors for the alteration of volatile compounds content.

## Conclusions and Perspectives

Present findings showed that soil-less farming, especially aeroponic cultivation is a promising and sustainable agro-practice to cultivate and produce year-round quality biomass as compared to conventional cultivation. The aeroponic system provides an easy-to-access subsequent roots harvest without sacrificing endangered plant species, and avoiding incidences of diseases. Besides roots, leaves could also be utilized as a prominent source for valerenic acid and its derivatives. Consequently, this system could be suitable for those plants that have volatile metabolites in their aerial parts. Further research is also needed to determine the volatile leakage or secondary metabolite losses in the aeroponic system for root medicinal plants, particularly. The current investigation also opens an avenue to meet the industrial standard for quality products; as no morpho-physiological aberrations were observed, in terms of plant growth and development. Elicitation mediated aeroponic cultivation for targeted metabolites enhancement in medicinal plants might be exploited for its pharmaceutical applications and commercial-scale production in less duration to meet the unmet demand of the industries. In conclusion, research investigation showed a significant contribution towards modernizing the conventional agricultural practices, for sustainably catalyzing the bio-economy to strengthen the farming community as well as for industrial usage.

## Data Availability Statement

The raw data supporting the conclusions of this article will be made available by the authors, without undue reservation.

## Author Contributions

AW, MP, and PK: Conceived the concept and framed the experimental design. AW, MP, and PK: Data taking and statistical analysis. AW, MP, PK, AK, RJ, and DK: Phytochemical analysis. MP, PK, AW, and DK: Manuscript writing and editing.

## Conflict of Interest

The authors declare that the research was conducted in the absence of any commercial or financial relationships that could be construed as a potential conflict of interest.
