# Supplementary material for: Effect of Elicitors on Morpho-Physiological Performance and Metabolites Enrichment in Valeriana jatamansi Cultivated Under Aeroponic Conditions
Source: Front Plant Sci. 2020 Sep 30;11:01263. doi: 10.3389/fpls.2020.01263 (PMC7561395; doi:10.3389/fpls.2020.01263)
Supplement: Supplementary file 1 [file Table_1.doc]

**Supplementary information for**

# “**Effect of elicitors on morpho-physiological performance and metabolites enrichment in *Valeriana jatamansi* cultivated under aeroponic conditions**”

*Mahinder Partap^1, 2, #^, Pankaj Kumar^1, #^, Anil Kumar^3^, Robin Joshi^4^, Dinesh Kumar^3*^**and Ashish R Warghat^1*^*

*^1^Cell and Tissue Engineering Laboratory, Biotechnology Division, Council of Scientific and Industrial Research - Institute of Himalayan Bioresource Technology, Palampur-176061, Himachal Pradesh, India*

*^2^Academy of Scientific and Innovative Research, Ghaziabad, Uttar Pradesh-201002, India*

*^3^Natural Product Chemistry and Process Development Division, Council of Scientific and Industrial Research -Institute of Himalayan Bioresource Technology, Palampur-176061, Himachal Pradesh, India*

*^4^Biotechnology Division, Council of Scientific and Industrial Research -Institute of Himalayan Bioresource Technology, Palampur-176061, Himachal Pradesh, India*

*# Authors contributed equally to this work and considered as joint first author*

*^*^****Correspondence email:***[*ashishwarghat@ihbt.res.in*](mailto:ashishwarghat@ihbt.res.in) *(AW)*; [*dineshkumar@ihbt.res.in*](mailto:dineshkumar@ihbt.res.in) *(DK)*

The supporting supplementary file gives an information about parameters optimization in aeroponic system represented in figure S1. UPLC chromatograms of standards and samples represented in table S1 and figure S2 to S31 as given below.


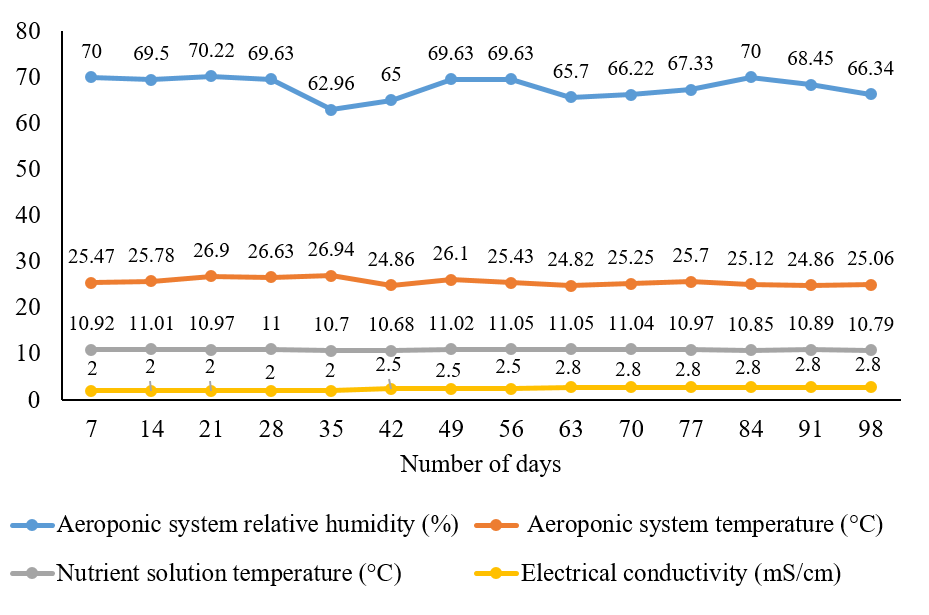


**Figure S1** Parameters optimization (relative humidity, system temperature, nutrient solution temperature and electrical conductivity) for *V. jatamansi* cultivated under aeroponic system.

**Table S1** Standard calibration curves and chromatogram of valerenic acid and its derivatives by UPLC method.

| Standard compounds | Acetoxy valerenic acid (AVA) | Hydroxy valerenic acid (HVA) | Valerenic acid (VA) |
| --- | --- | --- | --- |
| Calibration curves | 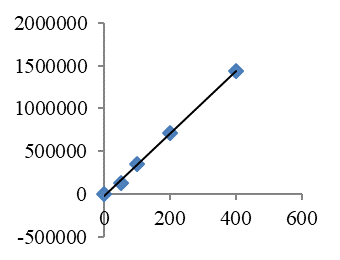 | 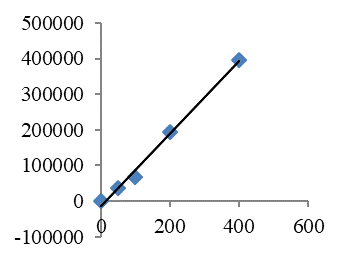 | 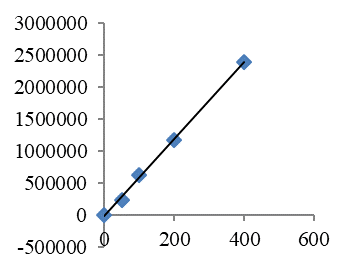 |
| Linearity range (µg/mL) | 50 – 400 | 50 – 400 | 50 - 400 |
| Regression equation | *y* = 3653.2*x* - 19284 | *y* = 1019.1*x* – 14021 | *y* = 6026.8*x* - 21340 |
| Correlation coefficient (*r^2^*) | 0.999 | 0.995 | 0.999 |
| Standard Chromatogram | 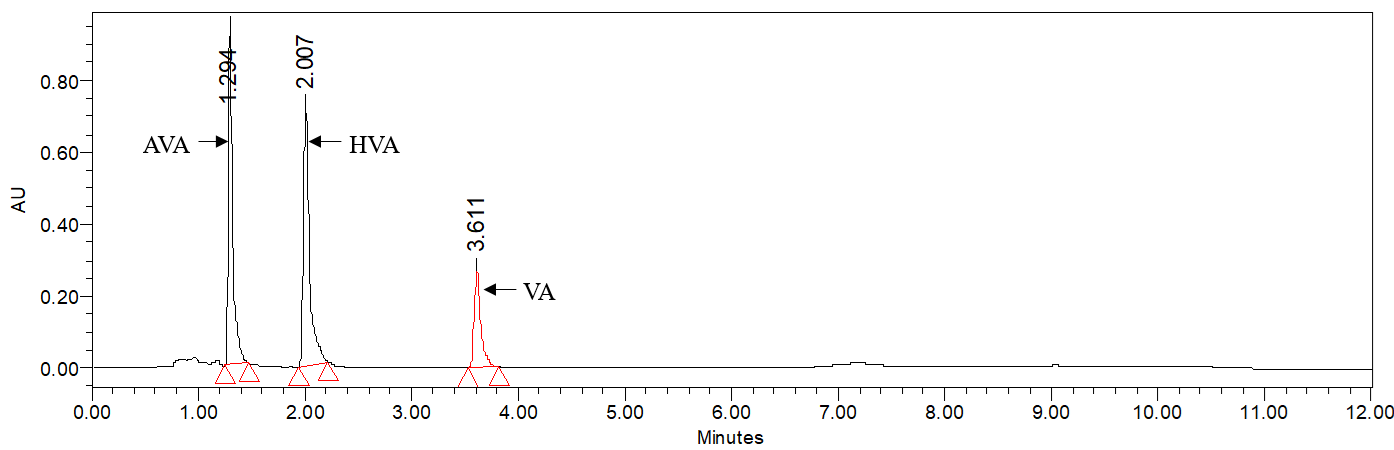 | | |

**Figure S2** Nursery grown leaf

**Figure S3** Nursery grown root

**Figure S4** Leaf (Aeroponic control)

**Figure S5** Root (Aeroponic control)

**Figure S6** Yeast extract 0.5 mg/L (Aeroponic leaf)

**Figure S7** Yeast extract 0.5 mg/L (Aeroponic root)

**Figure S8** Yeast extract 1.0 mg/L (Aeroponic leaf)

**Figure S9** Yeast extract 1.0 mg/L (Aeroponic root)

**Figure S10** Yeast extract 1.5 mg/L (Aeroponic leaf)

**Figure S11** Yeast extract 1.5 mg/L (Aeroponic root)

**Figure S12** Methyl jasmonate 50 µM (Aeroponic leaf)

**Figure S13** Methyl jasmonate 50 µM (Aeroponic root)

**Figure S14** Methyl jasmonate 100 µM (Aeroponic leaf)

**Figure S15** Methyl jasmonate 100 µM (Aeroponic root)

**Figure S16** Methyl jasmonate 150 µM (Aeroponic leaf)

**Figure S17** Methyl jasmonate 150 µM (Aeroponic root)

**Figure S18** Leaf (Pot control)

**Figure S19** Root (Pot control)

**Figure S20** Yeast extract 0.5 mg/L (Pot leaf)

**Figure S21** Yeast extract 0.5 mg/L (Pot root)

**Figure S22** Yeast extract 1.0 mg/L (Pot leaf)

**Figure S23** Yeast extract 1.0 mg/L (Pot root)

**Figure S24** Yeast extract 1.5 mg/L (Pot leaf)

**Figure S25** Yeast extract 1.5 mg/L (Pot root)

**Figure S26** Methyl jasmonate 50 µM (Pot leaf)

**Figure S27** Methyl jasmonate 50 µM (Pot root)

**Figure S28** Methyl jasmonate 100 µM (Pot leaf)

**Figure S29** Methyl jasmonate 100 µM (Pot root)

**Figure S30** Methyl jasmonate 150 µM (Pot leaf)

**Figure S31** Methyl jasmonate 150 µM (Pot root)
